# Effect of Bile Salt Hydrolase Inhibitors on a Bile Salt Hydrolase from *Lactobacillus acidophilus*

**DOI:** 10.3390/pathogens3040947

**Published:** 2014-12-17

**Authors:** Jun Lin, Rekek Negga, Ximin Zeng, Katie Smith

**Affiliations:** Department of Animal Science, The University of Tennessee, Knoxville, Tennessee, TN, 37996, USA; E-Mails: rnegga@utk.edu (R.N.); xzeng3@utk.edu (X.Z.); ksmit186@utk.edu (K.S.)

**Keywords:** antibiotic growth promoter, alternative, bile salt hydrolase, inhibitor, high-throughput screening, probiotics

## Abstract

Bile salt hydrolase (BSH), a widely distributed function of the gut microbiota, has a profound impact on host lipid metabolism and energy harvest. Recent studies suggest that BSH inhibitors are promising alternatives to antibiotic growth promoters (AGP) for enhanced animal growth performance and food safety. Using a high-purity BSH from *Lactobacillus salivarius* strain, we have identified a panel of BSH inhibitors. However, it is still unknown if these inhibitors also effectively inhibit the function of the BSH enzymes from other bacterial species with different sequence and substrate spectrum. In this study, we performed bioinformatics analysis and determined the inhibitory effect of identified BSH inhibitors on a BSH from *L. acidophilus*. Although the *L. acidophilus* BSH is phylogenetically distant from the *L. salivarius* BSH, sequence analysis and structure modeling indicated the two BSH enzymes contain conserved, catalytically important amino residues and domain. His-tagged recombinant BSH from *L. acidophilus* was further purified and used to determine inhibitory effect of specific compounds. Previously identified BSH inhibitors also exhibited potent inhibitory effects on the *L. acidophilus* BSH. In conclusion, this study demonstrated that the BSH from *L. salivarius* is an ideal candidate for screening BSH inhibitors, the promising alternatives to AGP for enhanced feed efficiency, growth performance and profitability of food animals.

## 1. Introduction

The food animal industry has manipulated gut microbiota to increase body weight and feed efficiency through the use of sub-therapeutic level of antibiotics, called antibiotic growth promoters (AGP), as feed additives for more than 50 years [1]. However, AGP usage has been linked to the emergence of antibiotic resistant bacteria [2]. Thus, there is a worldwide trend to limit AGP use in food animals to protect public health and food safety, which have created challenges for the animal industries [2,3]. Effective alternatives to AGP are urgently needed to maintain current animal production levels without threatening public health.

Examining the effect of AGP on intestinal microbiota in food animals would improve our understanding on the mode of action of AGP and facilitate the development of novel alternatives to AGP. Although reduction of gut pathogens due to AGP usage is potentially a mechanism contributing to growth promotion, it has been widely accepted that use of AGP would restructure the complex gut microbial for optimal host growth performance from nutrition standpoint. Recent independent food animal studies [4,5,6,7] have shown that the growth-promoting effect of AGP was highly correlated with the decreased activity of bile salt hydrolase (BSH), a gut bacterial enzyme that has negative impact on host fat digestion and energy harvest [8,9]. Notably, using both gnotobiotic and conventionally raised mice, Joyce *et al.* [10] recently have obtained direct supporting evidence demonstrating that BSH activity, the widely distributed function of the gut microbiota, significantly influences host lipid metabolism and weight gain. Based on these extensive supporting evidence, we have proposed that BSH is a promising microbiome target for developing novel alternatives to AGP; specifically, BSH inhibitors are promising feed additives to replace AGP for enhanced host lipid metabolism and growth performance [11].

The BSH enzyme produced by gut bacteria catalyzes deconjugation of conjugated bile acids, an essential gateway reaction in the metabolism of bile acids [8]. The natural functions of this BSH-mediated metabolic activity in the producing bacteria are still not clear despite various theories with contradictory findings [8]. However, it has been increasingly recognized that intestinal BSH plays an important role in host metabolism and energy harvest [8,10,11,12]. Because conjugated bile acids function as a more efficient “biological detergent” than unconjugated bile acids to emulsify and solubilize lipids for fat digestion [8], BSH activity has significant impact on host physiology by disturbing fat digestion and lipid metabolism, consequently affecting body weight gain [8,10,12]. Recently, we have identified and characterized a powerful BSH enzyme with broad substrate specificity from a chicken *Lactobacillus salivarius* strain [13]. In addition, with the aid of the purified *L. salivarius* BSH, we have identified a panel of BSH inhibitors using targeted screening [13] as well as high-throughput screening [14]. The *L. salivarius* BSH displayed potent hydrolysis activity towards both glycoconjugated and tauroconjugated bile salts; the broad substrate specificity nature of this BSH may make it an ideal candidate for screening desired BSH inhibitors targeting various BSH enzymes [13,14].

However, given different types of BSH enzymes present in the intestine [8,12], a significant question is raised: can these identified inhibitors also effectively inhibit the function of the BSH from other bacterial species with significant sequence variation and substrate spectrum? Addressing this issue is critical for us to identify desired BSH inhibitors using the established *L. salivarius* BSH-based high-throughput screening system [14]. In this study, we performed comparative genomic, structural and biochemical analysis of a BSH from a different strain *L. acidophilus*. The inhibitory effect of previously identified BSH inhibitors on the purified BSH from different species was determined.

## 2. Results and Discussion

In this study, we chose the BSH from *L. acidophilus* PF01 [15] for validation work due to following several reasons. First, compared to the *L. salivarius* BSH enzyme that used for screening BSH inhibitors [13,14], this BSH enzyme is produced by a different bacterial species. Second, the BSH-producing *L. acidophilus* PF01 and *L. salivarius* NRRL B-30514 strains were originally isolated from the intestine of two different food animals, swine and chicken, respectively. Finally, the *L. acidophilus* BSH (316 amino acids, aa) and the *L. salivarius* BSH (324 aa) displayed significant sequence variation (only 35% aa identity) and different substrate specificity [13,15]. Therefore, these differences make the *L. acidophilus* BSH an appropriate candidate enzyme to determine if previously identified BSH inhibitors [13,14], which is based on the *L. salivarius* BSH, could effectively inhibit the activity of diverse BSH enzymes in the intestine.

### 2.1. Phylogenetic and Structural Analysis of BSH

**Figure 1:**
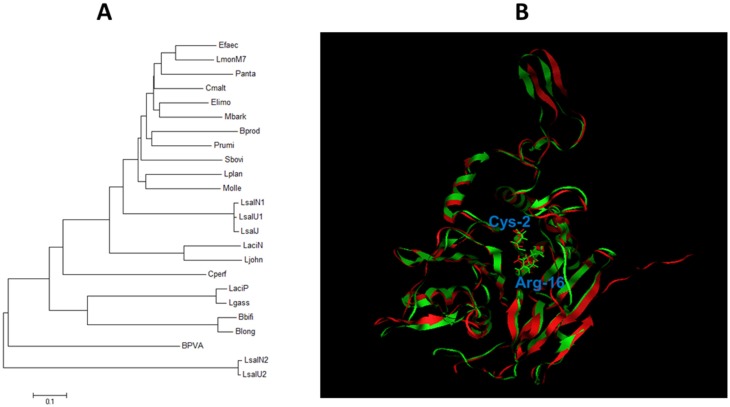
Sequence and structural analysis of bile salt hydrolase (BSH). (**A**) Phylogenetic relationship of BSH from different bacteria. The amino acid-based dendrogram was constructed in MEGA 6.0 by using neighbor-joining methods. LaciP, *L. acidophilus* PF01 (ABQ01980.1); LsalN1, *L. salivarius* NRRL B-30514 BSH1 (JX120368); LsalN2, *L. salivarius* NRRL B-30514 BSH2 (JX120369); LsalU1, *L. salivarius* UCC118 BSH1(ACL98201.1); LsalU2, *L. salivarius* UCC118 BSH2 (ABD99327.1); LsalJ, *L. salivarius* JCM1046 BSH1 (ACL98203.1); LaciN, *L. acidophilus* NCFM (AAV42923.1); Lgass, *Lactobacillus gasseri* (EFQ47028.1); Ljohn, *L. johnsonii* (EGP12391.1); Lplan, *L. plantarum* (AAA25233.1); Bbifi, *B. bifidum* (AAR39435.1); Blong, *B. longum* (AAF67801.1); Efaec, *Enterococcus faecium* (AAP20760.1); LmonM7, *Listeria monocytogenes* M7(AEH93162.); Cperf, *Clostridium perfringens* (AAC43454.1); BPVA, *Bacillus Sphaericus* PVA (YP_001698896). The number in parentheses is GenBank accession number. Furthermore, the following sequences of BSH homologs were extracted from IMG database (https://img.jgi.doe.gov/cgi-bin/w/main.cgi) based on similarity (>30%): Elimo, *Eubacterium limosum* KIST612; Bprod, *Blautia producta* ATCC 27340; Sbovis, *Streptococcus bovis* SN033; Panta, *Planococcus antarcticus* DSM 14505; Mbark, *Microbacterium barkeri* 2011-R4; Prumi, *Pseudobutyrivibrio ruminis* HUN009; Cmalt, *Carnobacterium maltaromaticum* MX5; Molle, *Methanobrevibacter olleyae* DSM 16632. (**B**) Structural modeling of BSH. Using *C. perfringens* BSH as a template, the structures of *L. acidophilus* BSH (green backbone) and *L. salivarius* BSH (red backbone) were predicted and superimposed. The RMSD value is 2.749 A. The side chains of critical residues C2 and R16 were denoted.

The complete BSH genes from diverse bacteria species were retrieved from database for analysis. As shown in Figure 1A, the BSH produced by *L. acidophilus* PF01 (LaciP) shared high homology (93% aa identity) to a BSH from *L. gasseri* (Lgass) but is phylogenetically distant from the BSH identified in many other bacteria, such as the BSH from *L. salivarius* NRRL B-30514 (LsalN1). Although the BSH enzymes from various bacterial species showed significant sequence variation (Figure 1A), multiple sequence alignment indicated that these BSH enzymes contain all conserved, catalytically important aa residues in the proposed active site of BSH (Cys-2, Arg-16, Asp-19, Asn-79, Asn-171, and Arg 224) [8] (Data not shown). This conservativeness of catalytically important motifs suggests that previously identified BSH inhibitors may effectively inhibit diverse BSH enzymes.

We also performed structural modeling of the *L. acidophilus* BSH (LaciP) and the *L. salivarius* BSH (LsalN1) by using the only known crystal structure of the *Clostridium perfringens*-produced BSH [16] (Cperf in Figure 1A), which indicated that the *L. acidophilus* BSH and the *L. salivarius* BSH shared similar structure by showing the typical canonic αββα-folding pattern (Figure 1B). Consistent with the structural similarity between the two different BSH enzymes (Figure 1B), the critical amino acids are also superimposed very well, particularly with respect to the typical Cys2, which served as an N-terminal nucleophile, and the Arg16, which play a potentially essential role in catalytic functioning of the enzyme [16]. This structure modeling provides further evidence supporting the feasibility of using the *L. salivarius* BSH for screening desired BSH inhibitors.

### 2.2. Expression and Purification of L. acidophilus Recombinant BSH (rBSH)

**Figure 2:**
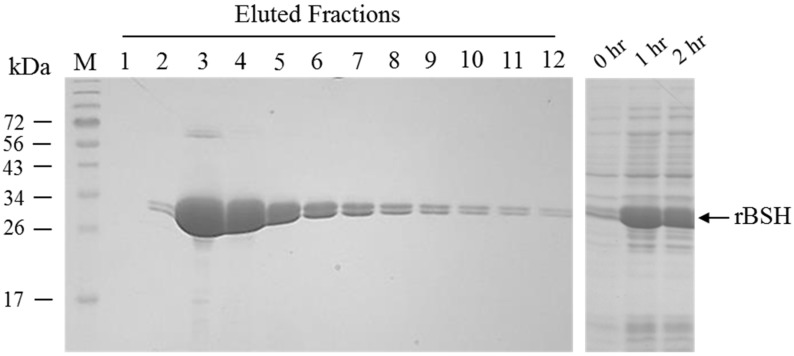
Production of purification of the *L. acidophilus* BSH enzyme. Lane M, EZ Run^TM^ prestained molecular mass marker (Fisher Bioreagent); Lane 1–12, eluted fractions during Ni-nitrilotriacetic acid affinity chromatography purification; 0 h, whole-cell lysate of noninduced *E. coli.*; 1 h, whole-cell lysate of *E. coli* induced with 0.5 mM IPTG for 1 h; 2 h, whole-cell lysate of *E. coli* induced with 0.5 mM IPTG for 2 h.

A pET-21b(+) vector bearing the full length of a *L. acidophilus* BSH gene was transformed to an *E. coli* BL21 (DE3) host strain for production of recombinant BSH (rBSH). Upon induction by 0.5 mM of IPTG for as short as 1 h, the recombinant *E. coli* construct significantly produced an additional protein with approximate molecular mass of 32 kDa on SDS-PAGE, consistent with the calculated molecular mass from the deduced amino acid sequence of the rBSH (Figure 2). The high-purity of the C-terminal His-tagged rBSH was subsequently obtained from the *E. coli* culture using one-step Ni-NTA agarose affinity chromatography. As shown in Figure 2, the high-purity of rBSH was predominantly present in the eluted fractions number 3 to 6. Interestingly, a band with slightly smaller molecular mass was co-present with the rBSH; this band likely represents a partially degraded rBSH (Figure 2). Approximately 25 mg of the rBSH was consistently purified from 1 liter of induced culture.

### 2.3. The Identified BSH Inhibitors also Inhibited the Activity of the L. acidophilus BSH

As shown in Table 1, almost all of previously identified BSH inhibitors using the *L. salivarius* BSH [13,14] also exerted potent inhibitory effect on the phylogenetically distant BSH produced by *L. acidophilus*, which strongly supported our hypothesis that the *L. salivarius* BSH is an ideal candidate for screening desired BSH inhibitors targeting various BSH enzymes in the intestine. As expected, only limited BSH inhibitors, such as ZnSO_4_ and roxarsone, displayed weaker inhibitory effect for the *L. acidophilus* BSH (Table 1) when compared to the *L. salivarius* BSH [14], which is likely caused by sequence-related subtle changes in catalytic motif region and structure. Based on these findings, the *L. salivarius* BSH in conjunction with an efficient high-throughput screening system [14] would serve as a solid platform for us to identify desired BSH inhibitors with potential to replace AGP for enhanced host lipid metabolism and growth performance.

**Table 1:** Effect of identified BSH inhibitors on the activity of the *L. acidophilus* BSH enzyme.

Compound Category	BSH Inhibitor	% Inhibition
^a^ The approved feed additives used in food animal industry [13]	KIO_3_	99.1
	NaHIO_3_	99.3
	NaIO_4_	99.0
	CuSO_4_	94.7
	CuCl_2_	97.2
	ZnSO_4_	27.4
	ZnCl_2_	38.4
^b^ The novel BSH inhibitors identified using high-throughput screening [14]	Menadione	97.9
	Riboflavin ^c^	96.5
	Gossypetin	96.1
	Caffeic Acid Phenethyl Ester (CAPE)	71.8
	Epicatechin monogallate	52.8
	Purpurogallin	36.1
^d^ The antibiotics that can inhibit BSH activity [14]	Oxytetracycline	99.6
	Demeclocycline Hydrochloride	99.6
	Methacycline Hydrochloride	99.2
	Doxycycline Hydrochloride	98.3
	Roxarsone	48.6
	Lincomycin	26.8

^a^ The final concentration of dietary compound in the reaction mix was 5 mM to achieve optimal resolution with the quantitative BSH activity assay; ^b^ Unless specified, the final concentration of specific BSH inhibitor was 2.5 mM; ^c^ The final concentration of riboflavin in reaction mix was 0.5 mM; ^d^ The final concentration of specific antibiotic was 2.5 mM.

**Figure 3:**
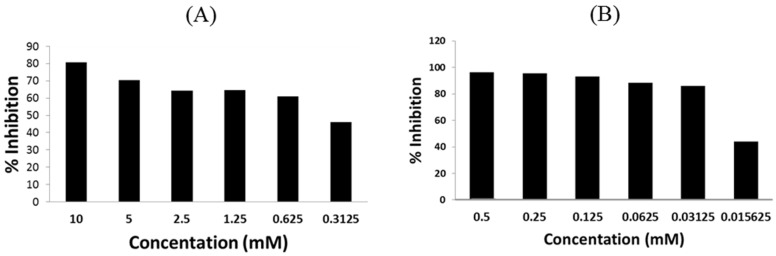
Dose-dependent effects of selected BSH inhibitors on the activity of the *L. acidophilus* BSH. (**A**) Inhibition of BSH activity by Caffeic Acid Phenethyl Ester (CAPE). (**B**) Inhibition of BSH activity by riboflavin.

Our recent study [14] has suggested two novel BSH inhibitors, riboflavin and caffeic acid phenethyl ester (CAPE), have high potential as novel alternative to AGP. Riboflavin is a vitamin that has been used as feed additive in poultry to treat the hypovitaminosis B2. However, long-term dietary supplementation of riboflavin for growth promotion in broilers has never been explored. Notably, a recent swine study has showed that the dietary supplementation of high-level riboflavin (20 mg/kg feed) significantly increased feed efficiency and body weight gain in the pigs with high lean growth although underlying mechanisms are still not clear [17]. CAPE has antioxidant/anti-inflammatory effects and are emerging natural food additive that recently has drawn extensive attention for human and animal application. In this study, we also observed potent inhibitory effect of these two compounds on the *L. acidophilus* BSH in this study (Table 1). Since we are particularly interested in CAPE and riboflavin as AGP alternatives, subsequent dosing experiments were conducted to examine if they could inhibit BSH activity at lower concentrations. As shown in Figure 3, CAPE still inhibited rBSH activity by more than 50% at a final concentration of 0.625 mM (Figure 3A) and riboflavin by more than 50% at a final concentration as low as 0.03125 mM (Figure 3B). Together the findings from this study and previous study [14] highly warrant future comprehensive animal trials to determine the effects of dietary supplementation of these two novel BSH inhibitors on growth performance and host lipid metabolism.

Interestingly, our recent high-throughput screening work [14] also identified a panel of antibiotics as BSH inhibitor, including a panel of tetracycline antibiotics and roxarsone that have been used as AGPs in food animals. This surprising finding suggests a new mode of action of AGP by direct inhibition of intestinal BSH enzymes for enhanced lipid metabolism and energy harvest. In this study, we also observed that the antibiotics, particularly those belonging to tetracycline class, also exerted potent inhibitory effect on the *L. acidophilus* BSH (Table 1). Clearly, the compounds belonging to an antibiotic family will not be considered as potential alternatives to AGP in the future. However, examination of the BSH inhibitors belonging antibiotic group here further supported our hypothesis and also provided new insights into the mode of action of AGP.

## 3. Materials and Methods

Bioinformatics analysis of BSH. A BSH gene from *L. acidophilus* PF01 has been identified and characterized by Hae-Keun *et al.* [15]; the nucleotide sequence of this BSH gene was deposited in the GenBank database (Accession No. EF536029). The BSH gene from the *L. acidophilus* PF01 strain [15] were compared to those identified from *L. salivarius* NRRL B-30514 [13] and from other diverse bacteria using the BLASTP program from the National Center for Biotechnology Information (NCBI, http://www.ncbi.nlm.nih.gov/). To reveal the phylogenetic relationship, multiple sequence alignment of BSH sequences from different bacterial species and penicillin V acylase from *Bacillus sphaericus* (BPVA) was performed using the ClustalW program in MEGA 6.0 [18]. The dendrogram was constructed by neighbor-joining methods. To identify the conserved amino acid motifs potentially involved in BSH activity, multiple sequence alignment of BSH enzymes was performed using the ClustalW2 program.

The modeling of BSH was performed in the Molecular Operating Environment (MOE), version 2008.10 (Chemical Computing Group, Montreal, QC). The BSH from *Clostridium perfringens* [16] was chosen as template. Total 10 models were generated in the MOE homology module, using the AMBER99 force field. The one with highest packing score was chosen to superimpose with the *C. perfringens* BSH [16], using the substitution matrix blosum62.

Purification of recombinant BSH (rBSH). A pET-21b (+) vector-derived recombinant plasmid encoding recombinant *L. acidophilus* BSH [15] was kindly provided by Dr. Dae-Kyung Kang (Dankook University, Korea). This recombinant plasmid bears a histidine-tagged rBSH gene with a full-length of BSH gene cloned from *Lactobacillus acidophilus* PF01, a commensal strain isolated from swine intestine [15]. In this study, this recombinant plasmid was introduced into the *E. coli* BL21 (DE3) host strain via transformation and desired transformants were selected on Luria-Bertani (LB) agar plates supplemented with ampicilin (100 μg/mL) following overnight incubation at 37 °C. The recombinant plasmid in one transformant, designated as JL1139, was extracted and subsequently sequenced; no mutations in the coding sequence of the BSH gene were detected. Expression and purification of the His-tagged rBSH from JL1139 were performed using the procedure described in previous publications [13,15,19]. Sodium dodecyl sulfate-polyacrylamide gel electrophoresis (SDS-PAGE) with a 12% (wt/vol) polyacrylamid separating gel was performed to monitor production and purification of the rBSH. The purified rBSH was finally dialyzed against PBS buffer containing 10% of glycerol (pH 7.0) and stored in −80 °C freezer prior to use. Protein concentration was measured by BCA protein assay kit (Pierce).

Effect of identified BSH inhibitors on the activity of BSH. The following three groups of compounds that have been identified as inhibitors for the *L. salivarius* BSH [13,14] were used in standard BSH assay in this study: (1) the approved feed additives used in food animals including CuCl_2_, CuSO_4_, ZnCl_2_, ZnSO_4_, NaHIO_3_, KIO_3_ and NaIO_4_; (2) the novel BSH inhibitors identified using high-throughput screening, which include caffeic acid phenethyl ester, riboflavin, epicatechin monogallate, gossypetin, menadione, and purpurogallin [14]; (3) the antibiotics that can inhibit BSH activity including oxytetracycline, demeclocycline hydrochloride, methacycline hydrochloride, doxycycline hydrochloride, roxarsone, and lincomycin [14].

A modified two-step standard BSH assay [13] was performed to determine the inhibitory effect of the selected BSH inhibitors on the activity of the rBSH from L. acidophilus. Breifly, 10 µL of specific inhibitor,10 µL of rBSH (1.20 µg/µL), 168 µL of reaction buffer (0.1 M sodium-phosphate, pH 6.0) and 2 μL of 1 M DTT were mixed gently and incubated at 37 °C for 30 min. Then 10 μL of glycocholic acid (100 mM) was added in the 190 μL of reaction mix and the final reaction mix (total volume of 200 µL) was incubated at 37 °C for another 30 min. A 50-μL aliquot of the reaction mixture was then immediately mixed with 50 μL of 15% (w/v) trichloroacetic acid for stopping the reaction, followed by centrifugation for 5 min at 12,000 × *g* at room temperature to remove the precipitate. The supernatant was used in the second step, in which 50 μL of supernatant was thoroughly mixed with 950 μL of ninhydrin reagent mix (0.25 mL of 1% [wt/vol] ninhydrin in 0.5 M Sodium-citrate buffer, pH 5.5; 0.6 mL of glycerol; and 0.1 mL of 0.5 M sodium-citrate buffer, pH 5.5). A positive control (with BSH enzyme, without BSH inhibitor) and a negative control without BSH added were set up in each independent experiment. All assays were performed in triplicate. Percentage inhibition was calculated by dividing the inhibited activity (mean activity of control—mean residual activity of presence of a compound) relative to the mean activity of control and then multiplied by 100.

## 4. Conclusions

BSH enzyme is a promising microbiome target for developing novel alternatives to AGPs to enhance the productivity and sustainability of food animals. This study demonstrated that recently developed high-throughput screening system using a *L. salivarius* BSH [14] is a feasible platform for us to discover BSH inhibitors that may target diverse BSH enzyme in the intestine. In the future, comprehensive animal trials are needed to determine the effects of dietary supplementation of BSH inhibitors on feed efficiency, lipid metabolism, and growth performance of food animals.
